# Across the Curious Parallel of Language and Species Evolution

**DOI:** 10.1371/journal.pbio.0060186

**Published:** 2008-07-22

**Authors:** John Whitfield

## Abstract

Evolutionary biologists are applying their tools to language--and trying to work out why the dynamics of language and biological evolution look so similar.

In February 1837—even before he sailed on the *Beagle*—Charles Darwin wrote to his sister Caroline, discussing the linguist Sir John Herschel's idea that modern languages were descended from a common ancestor. If this were really the case, it cast doubt on the Biblical chronology of the world: “[E]veryone has yet thought that the six thousand odd years has been the right period but Sir J. thinks that a far greater number must have passed since the Chinese [and] the Caucasian languages separated from one stock” [1].

The example of language change was a lifelong influence on Darwin's thought (see Figure 1). In *The Origin of Species*, he argued that our ability to order languages genealogically, despite their having changed and divided at different rates, shows that the same can be done for species [2]. And in *The Descent of Man*, he noted that: “The formation of different languages and of distinct species, and the proofs that both have been developed through a gradual process, are curiously parallel” [3].

**Figure 1:**
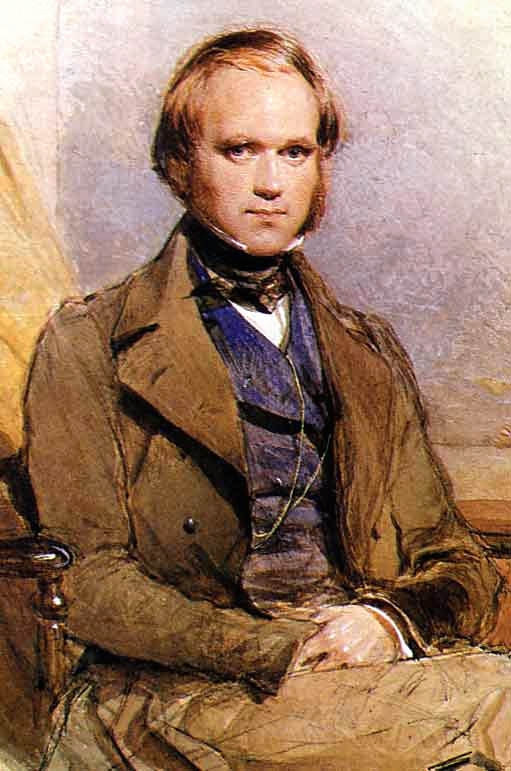
Language and Darwin The idea that modern languages had descended from a common ancestor, as proposed by Sir John Herschel, inspired the young Charles Darwin to think about the notion of descent with modification in relation to biological evolution. (Water-color portrait of Charles Darwin painted by George Richmond in the late 1830s.)

The tools of evolutionary analysis now allow both biologists and linguists to investigate whether these parallel paths might actually intersect, or perhaps be lanes of the same highway. And by giving the study of language change a quantitative edge, this approach has revealed striking similarities between the dynamics of biological evolution and language change. “Languages are extraordinarily like genomes,” says evolutionary biologist Mark Pagel of the University of Reading, UK. “We think there could be very general laws of lexical evolution to rival those of genetic evolution.”

What form this law might take is up for grabs; a particular mystery is how the regular changes that become apparent over centuries and millennia relate to the myriad processes that influence how individuals learn and use language. Evolutionary ideas are making their presence felt here, too, although the relative contribution of biological and cultural evolution, and how they might interact, is disputed. But it's possible, say some, that an understanding of how language changes could form part of a general theory encompassing both biological and cultural evolution. “If there's a model system for cultural evolution, then probably the people working on language have got it, because there's so much data,” says psychologist Alex Mesoudi of Cambridge University.

## Smooth, Yet Jerky

“Languages are extraordinarily like genomes…there could be very general laws of lexical evolution to rival those of genetic evolution.”

One parallel between living things and languages is that their most important components show the least variation. In biology, this means that genes such as those involved in the machinery of protein synthesis change so slowly that they can be used to discern the relationships of groups that diverged hundreds of millions of years ago. Likewise, the most commonly used words, such as numbers and pronouns, change the most slowly. Looking at 200 of the commonest words in 87 Indo-European languages, Pagel's team found that the frequency with which they are used in everyday speech explains 50% of the variation in the rate of word change [4]. Similarly, Erez Lieberman, an evolutionary theorist at Harvard University, and his colleagues have found that over the past millennium, English verbs have become regularized at a rate inversely proportional to their frequency [5]. The frequency effect means that some rates of lexical replacement are comparable to the evolutionary rates of some genes, says Pagel; he thinks that these words might allow researchers to build family trees showing the relationships between languages reaching back 20 millennia, compared with the 8,000 years or so that most linguists currently think possible.

Linguists had long argued for a link between frequency of use and rate of change. “Neither finding came as a great surprise,” says Tecumseh Fitch, a cognitive scientist at the University of St Andrews in Scotland. “But making the hypothesis explicit and testable is a big step forward.” And the pattern's strength was striking: “You get incredible regularity, and really law-like processes, with a very simple model,” comments psychologist Russell Gray of the University of Auckland in New Zealand, who has borrowed the techniques of molecular phylogenetics to investigate how languages are related. “It shows there's something constant and regular about how functional processes affect the rate of change of our vocabulary.”

Languages…change slowly for a long time, and then undergo a sudden burst of change—what biologists call punctuated equilibrium.

But not everything about language change is regular. Earlier this year, Pagel and his colleagues uncovered another parallel between linguistic and biological change. Languages, they found, change slowly for a long time, and then undergo a sudden burst of change [6]—what biologists call punctuated equilibrium. These bursts seem to coincide with periods of linguistic speciation, when populations split and their languages diverge. Looking at trees of Indo-European, Austronesian, and Bantu languages, the researchers found that those languages that had gone through the most splits had changed more, with up to a third of changes being associated with split points. Pagel suggests that languages change when populations split because groups consciously or unconsciously use how they talk to define themselves and separate insiders from outsiders—as in the Old Testament book of Judges, when the men of Gilead identify their Ephraimite foes by their inability to pronounce the Hebrew word for an ear of grain, *shibboleth*, now a general term for a linguistic password.

## Genes and Culture

On the one hand, language change shows regular, law-like features, while on the other hand, such changes happen in fits and starts, perhaps driven by the changing allegiances and social goals of its users. Not surprising, then, that there are lots of suspects for drivers of language change. Sociolinguists emphasize how individual choices can become population processes when, for example, people copy the majority around them, or imitate high-status individuals. Followers of Noam Chomsky focus on how children end up speaking their parents' tongue in a slightly different form. But no one theory has firm support, says linguist William Croft of the University of New Mexico in Albuquerque. “A lot of mechanisms have been kicked around, but it's difficult to demonstrate which ones operate in any particular situation.”

Recently, genetics has joined the list of possible influences on how languages change. Last year, Dan Dediu and Robert Ladd, two linguists working at the University of Edinburgh, published a paper showing that the geographical distribution of variant forms of two genes active during brain development, called *ASPM* and *Microcephalin*, correlates with the distribution of tonal languages, where the inflection of a word changes its meaning [7]. In places where the ancestral form of the genes is commonest, such as in Southeast Asia and sub-Saharan Africa, the languages, such as Chinese and Yoruba, tend to be tonal. Where the derived form predominates, such as in Europe, West Asia, and North Africa, the languages, such as Spanish and German, are nontonal.

“Cultural change and biological change share the same fundamental properties of variation, selection and inheritance.”

These aren't genes for speaking Chinese. Any child will pick up the tongue (or tongues) it hears most often during the critical period for language learning, regardless of its ancestry. Rather, Ladd speculates that the different forms of the gene “direct the cultural evolution of language over multiple generations” by causing differences in the brain structures that affect how people hear or speak language. “Languages evolve to suit the genetic makeup of their speakers,” says Ladd.

Some researchers believe that language adapts to the brains of its speakers more generally and that this, rather any special biological endowment, has been the dominant influence on the features of language. Psychologist Morten Christiansen at Cornell University in Ithaca, New York thinks that abstract knowledge of language cannot be innate, because natural selection results in adaptations tailored to local environments and would thus have created different biological endowments across different populations of language users. The fact that human populations dispersed quickly and widely but modern children can still learn all languages suggests that this is not the case, he argues. He also thinks that any genetically encoded grammatical rules would rapidly become obsolete and maladaptive, because language is a fast-moving target, changing orders of magnitude faster than genes [8]. “The fit between the mechanisms of learning and processing languages and the structures of languages themselves is better explained by how languages have changed to fit the brain over hundreds, perhaps thousands, of generations, rather than how the brain has changed to fit language,” says Christiansen. Findings such as the increasing regularity of English verbs “fit very nicely” with this view, he says. “It's a microcosm of one of the key processes of language evolution.”

Simon Kirby, also at the University of Edinburgh, thinks that the key biological attribute that allows humans to learn language might not be genetically encoded grammar but vocal learning—the ability to remember and reproduce sequences of sound, which is also seen in songbirds and bats. “We could just be a chimp that can sing,” he says. Kirby has built computer simulations and theoretical models showing that a population with no language can develop one by cultural evolution alone, if it has arbitrarily small and very general cognitive biases, such as constraints on memory. Such biases mean that each generation preferentially uses those aspects of its language that are easiest to learn. This process, Kirby argues, leads to many of the features of language commonly interpreted as reflecting biological specialization, such as the way that all languages break down utterances into words [9]. “If you have individuals learning from individuals over the generations, the basic properties of language fall out, without having to be built in,” he says.

In lab experiments, Kirby has asked subjects to learn a nonsense language and then teach it to new subjects, and so on. He found that the randomness quickly became regularized, as people unconsciously shaped words into something easier to remember and use, and devised rules to come up with words for things they hadn't seen. Such a process may be at work in the spontaneous emergence over the past few decades of two sign languages—Nicaraguan Sign Language and Al-Sayyid Bedouin Sign Language. Each of these has moved rapidly from a system of gestures to a fully fledged language with conventions for grammar and sentence structure. Kirby plans to use them as a test bed for his ideas about how structure in language can rapidly emerge.

Not surprisingly, the idea that human language reflects no special biological traits is controversial. Language is so unusual and important to humans, says Fitch, that to think it doesn't need biological specializations is a mistake. “There's very powerful selection on every generation of children to learn language quickly and effectively,” he says. “Languages will inevitably ‘adapt’ to their users, but Christiansen seems to think that biological evolution will cease, which is fundamentally erroneous. I think he's fallen into the trap of thinking that languages have an independent life of their own.”

## Merging the Paths

Of course, lots of things drive language change, on many timescales. Modern English might bear the stamp of millennia of subtle prompting from *ASPM* and *Microcephalin*, but it certainly reflects the French spoken by the less-then-subtle Norman invaders of 1066. “Language contact is a very powerful force in language change,” says Ladd—although the fact that it's still possible to draw phylogenetic trees of languages shows that this form of horizontal transfer does not entirely swamp change within lineages. Harvard's Lieberman suspects that ideas from many sources will be needed to explain language change. Theories from epidemiology and the study of networks could help explain how social contacts cause particular linguistic forms to spread, he suggests, and demography could be used to analyse how language passes down the generations, and through the centuries. “There's tremendous room to get into the mechanistic nitty-gritty, and a tremendous richness of timescales to study, but we'll need a range of tools,” he says. The same tools, says Lieberman, could be used to analyse how technologies such as the printing press or ideas such as democracy or religion spread and change over time. “Whenever one has something you can call a meme, there might be techniques for studying how that meme gets passed around,” he says.

Pagel suspects that language evolution is actually too similar to its biological counterpart to provide a general model for cultural change. “I don't think we'll learn a whole lot about cultural evolution generally from studying language evolution,” he says. “The heritability is too high, and the transmission too precise.” Fitch is more optimistic, pointing, again, to the wealth of data on language relative to our other cultural processes. “If there's ever to be a science of memetics, language will be the jewel in the crown,” he says.

Some researchers believe that cultural and biological evolution could be unified. Cultural and linguistic processes might be subject to forms of selection not seen in biology, says Croft, such as when people follow the leader or the majority [10]. But ultimately, these are all different versions of the same thing. “I have to constantly fend off the view that applying evolutionary ideas to linguistics is an analogy,” he says. “It's not an analogy: these are two different instantiations of a general theory of evolutionary change. These are early days, but such a theory will give us insights that you can't get just by looking at one domain.”

Mesoudi agrees: “Cultural change and biological change share the same fundamental properties of variation, selection and inheritance,” he says—adding that other processes, such as the workings of the immune system, or learning and memory, might follow the same rules. In a parallel universe, where Captain Fitzroy found someone else to accompany him on the *Beagle* and Darwin was left to pursue his linguistic musings, it might be the biologists looking to the social scientists for inspiration, he says. “There's no reason why the historical sequence shouldn't have been reversed, and the biologists were the ones catching up 100 years later.”
